# Outbreak of Fluoroquinolone-Resistant *Campylobacter jejuni* Infections Associated with Raw Milk Consumption from a Herdshare Dairy — Colorado, 2016

**DOI:** 10.15585/mmwr.mm6705a2

**Published:** 2018-02-09

**Authors:** Alexis Burakoff, Kerri Brown, Joyce Knutsen, Christina Hopewell, Shannon Rowe, Christy Bennett, Alicia Cronquist

**Affiliations:** ^1^Epidemic Intelligence Service, Division of Scientific Education and Professional Development, CDC; ^2^Colorado Department of Public Health and Environment, Denver, Colorado; ^3^Pueblo City-County Health Department, Pueblo, Colorado; ^4^El Paso County Public Health, Colorado Springs, Colorado; ^5^Division of Foodborne, Waterborne, and Environmental Diseases, National Center for Emerging and Zoonotic Infectious Diseases, CDC.

In August 2016, a local public health agency (LPHA) notified the Colorado Department of Public Health and Environment (CDPHE) of two culture-confirmed cases of *Campylobacter* infection among persons who consumed raw (unpasteurized) milk from the same herdshare dairy. In Colorado, the sale of raw milk is illegal; however, herdshare programs, in which a member can purchase a share of a herd of cows or goats, are legal and are not regulated by state or local authorities. In coordination with LPHAs, CDPHE conducted an outbreak investigation that identified 12 confirmed and five probable cases of *Campylobacter jejuni* infection. Pulsed-field gel electrophoresis (PFGE) patterns for the 10 cases with available isolates were identical using the enzyme *Sma*. In addition, two milk samples (one from the dairy and one obtained from an ill shareholder) also tested positive for the outbreak strain. Five *C. jejuni* isolates sent to CDC for antimicrobial susceptibility testing were resistant to ciprofloxacin, tetracycline, and nalidixic acid (*1*). Although shareholders were notified of the outbreak and cautioned against drinking the milk on multiple occasions, milk distribution was not discontinued. Although its distribution is legal through herdshare programs, drinking raw milk is inherently risky (*2*). The role of public health in implementing control measures associated with a product that is known to be unsafe remains undefined.

## Investigation and Results

On August 23, 2016, El Paso County Public Health notified CDPHE of two culture-confirmed cases of *C. jejuni* infection; campylobacteriosis is a reportable disease in Colorado. Both patients reported drinking unpasteurized milk from the same herdshare dairy in Pueblo County. Since 2005, obtaining raw milk by joining a herdshare program has been legal for Colorado residents, but selling raw milk is illegal. By purchasing a share of a herd (cows or goats), shareholders are entitled to a portion of the raw milk.

Because the prevalence of consuming unpasteurized milk is low (2.4% in Colorado, 2006–2007 FoodNet Population Survey; 3.1%, 2009 Colorado Behavioral Risk Factor Surveillance System), two cases of enteric illness with a common exposure to raw milk are unlikely to occur by chance (*3*,*4*). In this outbreak, a confirmed case was defined as diarrheal illness with onset on or after August 1, 2016, in a person with known consumption of unpasteurized milk from the same herdshare dairy and culture-confirmed *C. jejuni* infection. A probable case was defined as diarrhea onset on or after August 1, lasting 1 or more days, in a person with either known consumption of milk from the same herdshare dairy or with an epidemiologic link to a confirmed case.

Cases were identified through routine passive reporting with follow-up interviews, a Health Alert Network broadcast to area providers, and attempts to contact all shareholders. A public health order was issued to obtain a list of shareholders with their contact information after it was not provided by the dairy within 5 days of the initial request. CDPHE attempted to contact shareholders to inform them about the outbreak and assess possible illness. Up to three calls were made to each shareholder household. Epidemiologists contacted laboratories to request that isolates from potential outbreak-associated cases be forwarded to the state public health laboratory.

Among 91 (53%) of 171 shareholder households that responded to requests for follow-up interviews, representing 207 persons in five or more Colorado counties, 12 confirmed and five probable cases were identified (Figure). Among confirmed cases, patients ranged in age from 12 to 68 years (median = 58 years); nine were male. Duration of illness ranged from 3 to >10 days. One hospitalization occurred; there were no deaths. In addition to diarrhea, among the 12 confirmed cases, the majority of patients also experienced fever (10), abdominal pain or cramps (eight), headache (eight), and myalgia (seven); vomiting and bloody diarrhea were reported less frequently (in five and four persons, respectively).

**FIGURE:**
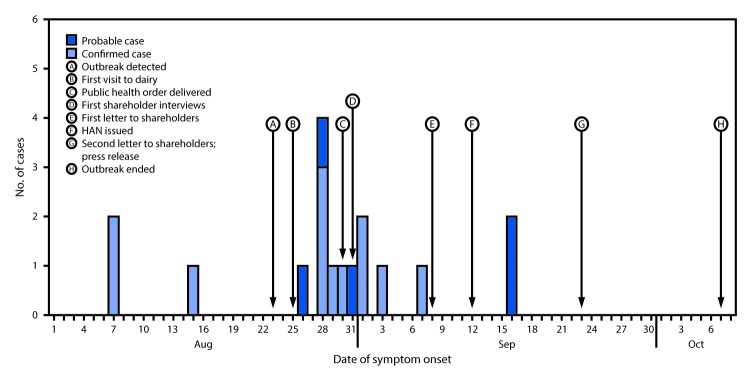
An outbreak of *Campylobacter jejuni* associated with consumption of raw milk from a herdshare dairy and public health response — Colorado, August 1–October 7, 2016 **Abbreviation:** HAN = Health Alert Network.

Four milk samples were tested for *C. jejuni*; pathogen identification and PFGE were performed on available isolates from persons epidemiologically linked to the outbreak. *C. jejuni* with one of two outbreak PFGE patterns (PulseNet DBRS16.0008 using the enzyme *Sma* and PulseNet DBRK02.1272 or DBRK02.0028 using the enzyme *Kpn*) was confirmed in 10 isolates that were available at the public health laboratory and two of the four raw milk samples. The National Antimicrobial Resistance Monitoring System performed antimicrobial susceptibility tests on five representative isolates; all were resistant to ciprofloxacin, tetracycline, and nalidixic acid (*1*).

## Public Health Response

Public health responses to this outbreak consisted of notifying shareholders about the outbreak on three occasions (Figure) and requiring the dairy to provide additional written notification about the outbreak at milk distribution points. A press release was issued by two LPHAs (Figure) in response to detecting at least one infection in a person who was not a shareholder but was given milk by shareholders. In addition, a number of shareholders reported sharing milk with nonshareholders who might have been unaware of the outbreak. Although milk sample results were positive for *C. jejuni*, CDPHE did not close the dairy or stop distribution of its milk because without pasteurization CDPHE could not create standards for safely reopening the dairy (*5*). Shareholders were, however, urged to discard raw milk distributed since August 1 and were reminded that Colorado statute prohibits redistribution of raw milk.

## Discussion

Raw milk from a herdshare dairy was the source of this outbreak of *C. jejuni* infections, and the investigation highlighted the difficulties inherent in addressing an outbreak related to unpasteurized milk from a herdshare dairy. During three previous herdshare-associated outbreaks in Colorado, public health authorities temporarily took action to stop milk distribution until a series of negative tests were obtained from the milk (Alicia Cronquist, CDPHE, personal communication, December 2017). However, because CDPHE could not ensure that unpasteurized milk would be safe in the future, the decision was made not to close the dairy during this outbreak. In addition, CDPHE’s Division of Environmental Health and Sustainability chose not to make formal recommendations on the dairy’s processes because no protocol improvements short of pasteurization could ensure the product’s safety, even with improved sanitation (*5*).

All tested isolates’ resistance to three antibiotics was concerning, particularly as fluoroquinolones are frequently used to treat *Campylobacter* infections in those cases where treatment is indicated. Treatment of antibiotic-resistant *Campylobacter* infections might be more difficult, of longer duration, and possibly lead to more severe illness than treatment of nonresistant *Campylobacter* infections (*6*–*8*). In 2015, approximately 25.3% of U.S. *C. jejuni* isolates were resistant to ciprofloxacin, an increase from 21.6% a decade earlier (*1*).

In collaboration with LPHAs, CDPHE is creating guidelines to address future outbreaks related to raw milk from herdshares. As more states legalize the sale or other distribution of unpasteurized milk, the number of associated outbreaks will likely increase (*9*,*10*). The role of public health in responding to raw milk–related outbreaks should be further defined. State-level guidelines might assist with this process.

**Summary**

**What is already known about this topic?**

Raw (unpasteurized) milk has been linked to many foodborne illnesses, including *Campylobacter* infections. In some states, including Colorado, it is legal to distribute unpasteurized milk through herdshare programs. Studies indicate that legalizing the sale of raw milk leads to more raw milk–associated outbreaks.

**What is added by this report?**

Although sale of raw milk is not legal in Colorado, herdshare programs, in which members may purchase a share of a herd of cows or goats, are legal and are not regulated by state or local authorities. During August–October 2016, 12 confirmed and five probable cases of *Campylobacter jejuni* infections were identified in persons who consumed raw milk from a herdshare dairy in Colorado. Pulsed-field gel electrophoresis identified the outbreak pattern in patients’ stools and two milk samples. Shareholders were notified about the outbreak, but the dairy was not ordered to close. This report highlights the public health challenges of addressing a high-risk product that is not regulated.

**What are the implications for public health practice?**

In states where distribution of raw milk from herdshares is legal, outbreaks associated with raw milk will likely continue to be a problem. The role of public health in implementing control measures associated with a product that is known to be unsafe should be further defined. State level guidelines might assist with this process.
